# Cerebral Small Vessel Disease Associated with Subclinical Vascular Damage Indicators in Asymptomatic Hypertensive Patients

**DOI:** 10.3390/bs9090091

**Published:** 2019-08-22

**Authors:** Zenaida Milagros Hernández-Díaz, Marisol Peña-Sánchez, Alina González-Quevedo, Sergio González-García, Paula Andrea Arias-Cadena, Marta Brown-Martínez, Mélany Betancourt-Loza, Anay Cordero-Eiriz

**Affiliations:** 1 Department of Neuroimagenology, International Center for Neurological Restoration, 25th Ave, Playa, 15805, Havana 11300, Cuba; 2 Neurobiology department, Neurology and Neurosurgery Institute, 29 St # 114, Plaza de la Revolución, Vedado, Havana 10400, Cuba; 3 Radiology Department, Clinical Nuestra-Cali/Colombia, 10 St # 33-51, Cali 760040, Colombia; 4 Clinical Neurophysiology department, Medicine Sport Institute, 10 St between 100 and 14, Embil, Boyeros, Havana 10800, Cuba

**Keywords:** cerebral small vessel disease, risk factors, essential hypertension, brain lesions, white matter hyperintensities

## Abstract

*Background*: Cerebral small vessel disease (CSVD) is frequent in patients with cardiovascular risk factors including arterial hypertension, and it is associated with vascular damage in other organs and the risk of stroke, cognitive impairment, and dementia. Early diagnosis of CSVD could prevent deleterious consequences. *Objective*: To characterize CSVD associated with indicators of subclinical vascular damage in asymptomatic hypertensive patients. *Materials and Methods*: Participants were hypertensive (HT) and non-hypertensive (non-HT) individuals; without signs of cerebrovascular disease, dementia, and chronic renal failure. For CSVD, white matter hyperintensities (WMH), enlarged Virchow–Robin perivascular spaces (EVRPS), lacunar infarcts, and microbleeds were investigated. Subclinical vascular damage was evaluated (hypertensive retinopathy, microalbuminuria, and extracranial carotid morphology: intima media thickness (IMT) and atheroma plaque). *Results*: CSVD MRI findings were more frequent in HT; as well as greater intimal thickening. The IMT and/or plaque was significantly associated with all MRI variables; but retinopathy was correlated with EVRPS and lacunar infarcts. Only microalbuminuria was related to the greater severity of WMH in HT. Multivariate analysis evidenced that CSVD was independently associated with the combination of indicators of vascular damage and systolic blood pressure. *Conclusions*: Combining indicators of subclinical vascular damage, such as carotid morphological variables, microalbuminuria, and hypertensive retinopathy for early detection of CSVD in asymptomatic hypertensive patients could prove to be useful to take actions for the prevention of irreversible brain damage, which could lead to cognitive impairment, dementia and stroke.

## 1. Introduction

Arterial hypertension (HT) is the most prevalent non-communicable disease worldwide, and has long been recognized as a major risk factor for cardiovascular diseases. It has a long-term unfavorable impact on target organs [1,2,3], worsens the process of atherosclerosis and increases the risk of acute myocardial infarction (AMI), heart failure [4,5], renal failure, and cerebrovascular disease (CVD) [6,7]. The effective control of cardiovascular risk factors and specifically blood pressure, decreases the incidence of these diseases; an aspect that should be considered in the treatment and prevention of its deleterious consequences [8].

The prevalence rate of HT in Cuba in 2009 was 202.7 per 1000 inhabitants, a value that has gradually increased to 219.4 in 2016 and 225.2 in 2017 [9,10]. Nevertheless, this value is probably underestimated according to the results of the National Survey for risk factors, which revealed that 33% of the population over 15 years of age is hypertensive [11], and this percentage increases with age and in subjects older than 60 years it reaches almost 80%.

Additionally, cardio and cerebrovascular diseases occupy the first and third causes of death respectively. In 2017, the mortality rates and the years of potentially lost life for these diseases increased. Heart conditions lead to 24,423 deaths in 2016 (217.4 per 100,000 inhabitants), increasing to 27,176 (241.6 per 100,000 inhabitants) in 2017; of these, 64.9% were of ischemic causes, and 45.3% of them due to AMI. On the other hand, 9 456 deaths due to CVD were reported in 2016 and 9913 in 2017, with rates of 84.2 and 88.1 per 100,000 inhabitants, respectively [10].

Therefore, early detection of subclinical brain damage is essential to reduce risks and prevent irreversible sequelae. Together with the heart, blood vessels, retina, and kidney, the identification of clinical or subclinical brain damage as a target organ is included within the stratification of cardiovascular risk, according to the European HT guidelines [12,13,14]. Since the brain is one of the most affected target organs in hypertension [15,16,17], early damage in the cerebral microcirculation leading to a microangiopathy—Called cerebral small vessel disease (CSVD)—is present [18,19]. These vascular lesions can be symptomatic or subclinical (“silent”) and have been related to essential HT in neurologically asymptomatic individuals [16,20,21,22,23]. In neuroimaging, these lesions can be observed as lacunar infarcts, microbleeds, white matter hyperintensities (WMH) and enlarged Virchow–Robin spaces (EVRPS) [18,19].

CSVD is five times more frequent than symptomatic CVD in the general population [24], and increase significantly with advancing age and HT [25,26]. These lesions are considered potential causes of disability [27,28], with unfavorable prognosis, and their presence predicts CVD and cognitive deterioration more frequently [20,21,23,25,29,30,31]. In fact, the annual cost, due to disability and rehabilitation for cardiovascular disease and stroke in the United States, has been estimated at 329.7 billion dollars [26].

Brain magnetic resonance imaging (MRI) has provided evidence of subclinical brain damage in asymptomatic subjects with comorbidities. Other more inexpensive and accessible biomarkers (clinical, imaging, molecular, and neurophysiological) have been explored with this objective, but studies are still inconclusive [20,21]. For this reason, the present investigation characterized CSVD in asymptomatic hypertensive patients and explored the relationship between CSVD and some indicators of subclinical vascular damage, as these indicators might be useful for screening hypertensive individuals, which are exposed to a higher risk of CVD, cognitive deterioration, and dementia.

## 2. Materials and Methods

An observational, descriptive, cross-sectional study with a case-control design was conducted in hypertensive patients (HT) and non-hypertensive individuals (non-HT). The subjects of both study groups came from the primary care level, from two health areas, “Moncada” Polyclinic, Plaza de la Revolución municipality, and “Cristobal Labra”, municipality of La Lisa, in Havana, Cuba. The study participants went to the Institute of Neurology and Neurosurgery between February 2015 and June 2018 to confirm the diagnosis of essential HT. Individuals with previous personal history of cardiovascular diseases (myocardial infarction or ischemic heart disease, and cardioembolic causes) and/or cerebrovascular diseases: cerebral infarcts (including lacunar infarcts of both carotid territories and symptomatic basilar artery), intraparenchymal hemorrhage, and transient ischemic attacks, as well as chronic renal failure were excluded. Additionally, patients with focal neurological signs, or those where ischemic heart disease, or other cardiac disease causing cardioembolism was suspected at clinical examination and confirmed after performing electro and/or echocardiogram, were also excluded.

Procedures:

All the subjects included in the study were subjected to a specialized neurological and general clinical examination. Additionally, brain MRI, echo-doppler of the supra-aortic trunk, fundoscopy, and electro and/or echocardiograms were performed in all subjects included. Blood chemistry and microalbuminuria were also assayed.

MRI studies were performed in a SIEMENS 3T Resonator at the Neurosciences Center, including T1-weighted images, FLAIR (fluid attenuated inversion recovery), T2, T2*, in 2 mm slices, sagittal, axial and coronal planes. In particular, WMH of vascular appearance were defined as hyperintense images in T2 and FLAIR, with absence of cavitation and were classified according to the modified Fazekas scale: 0: intensity employing Fazekas’ of periventricular and subcortical white matter; (I): soft halo surrounding the lateral ventricles or in a cap around the frontal horns and/or isolated hyperintensities in subcortical white matter; II: periventricular hyperintensities in thicker bands and/or subcortical hyperintensities that tend to converge; III: periventricular confluent lesions extending to the subcortical white matter. Lacunar infarctions were identified as cavities with fluid between 3 to 15 mm in diameter, located in the territories of perforating arterioles. They appear hypointense in T1 and hyperintense in T2; a hypointense cavity is seen in FLAIR sequence, surrounded by a hyperintense halo for reactive gliosis. EVRPS were considered as small well-defined images (smaller than 3 mm in diameter) with a signal intensity equal to the CSF located in basal ganglia, semioval center, or radiated corona, that followed the orientation of the perforating arteries. Microbleeds were identified as small hypointense lesions (2–5 mm in diameter, although sometimes up to 10 mm) in sequences of T2* [32].

The degree of WMH (hyperintense lesions of periventricular and subcortical white matter) was rated visually on axial FLAIR images and classified in two groups according to their intensity employing Fazekas’ score [20] as: 0–I (mild) and II–III (severe). Finally, the severity of CSVD was classified as mild when 0–1 vascular lesions were detected (lacunar infarctions, microbleeds, EVRPS, WMH), and severe when 2–4 vascular lesions were detected.

Doppler ultrasound protocol of the supra-aortic trunk was conducted displacing the transducer from the origin of the common carotid, to the most distal possible section of the external carotid and internal carotid. Use of color Doppler ultrasound, and subsequent recording of flow rates by pulsed Doppler were also performed; plaque morphology (if plaques were identified) was carefully evaluated and registered. Both carotid axes (common, external, and internal carotid on both sides) were explored employing an ALOKA brand PROSOUND alpha 10 scanner (Japan) equipped with a 7.5–13 MHz linear transducer, placed on each side of the patient’s neck. The following morphological variables were evaluated: intima media thickness (IMT), presence of plaques and the percentage of stenosis of the internal carotid artery. IMT was measured at the level of the common carotid artery, 1 cm from the bulb, and at the end of diastole. Intima media complex (IMC) thickening was considered when IMT > 0.9 mm. The presence of carotid atherosclerotic plaques was evaluated according to the Mannheim consensus: focal structure that invades the arterial lumen in at least 0.5 mm or 50% of the value of the surrounding intima media; or IMT > 1.5 mm measured from the adventitia interface to the intimal interface [33,34,35]. The combination of the variable IMT and/or plaque was made when the subjects presented at least one of these alterations.

In order to assess the degree of hypertensive retinopathy (HTRt) all subjects underwent a direct fundoscopic study, graded as: 0-normal, grades I–IV. Subjects with grade IV retinopathy were excluded because they did not meet the inclusion criteria [35,36].

Systolic and diastolic blood pressure (SBP and DBP respectively) were measured in all subjects with an aneroid sphygmomanometer according to the international recommendations of the US Seventh report of the Joint National Committee on Prevention, Detection, Evaluation, and Treatment of High Blood Pressure [37]. Blood pressure was measured at consultation when the patients were evaluated by the clinician, prior to ultrasound and MRI studies, and were averaged.

During the process of exclusion, echocardiographic study was performed with a conventional M-mode echocardiograph (PROSOUND ALOKA Alpha 10). Thickness of the interventricular septum (IVS) and the rear wall (RW) were measured. Reference values were: IVS < 11 mm and RW < 7–11 mm. Diastolic dysfunction was recorded as Yes or No. In addition, electrocardiograms were performed employing a CARDIOCID BB digital electrocardiograph with 12 derivations (COMBIOMED, Havana, Cuba).

Vascular risk factors were recorded in all subjects as follow: arterial hypertension when blood pressure (BP) readings ≥ 140/90 and/or with antihypertensive treatment. Diabetes mellitus (DM): subjects with one fasting glycemia ≥ 11.1 mmol/L, or two consecutive fasting glucose readings ≥ 7 mmol/L, or those receiving hypoglycemic medication (oral or insulin). Dyslipidemia: subjects with total cholesterol ≥ 5.5 mmol/L, or triglycerides ≥ 1.7 mmol/L, or receiving statin treatment. Obesity: subjects with a body mass index (BMI) greater than 30 kg/m^2^. The BMI was calculated according to the formula: BMI = Weight (kg)/[height (m)]^2^. Smokers and former smokers (until five years ago): subjects who smoked one or more cigarettes per day during at least one year prior to inclusion. Age over 55 years, according to the European guidelines arterial hypertension [38]. Finally, familial pathological background of cardiovascular and/or CVD.

Peripheral venous blood was extracted (10 mL) and collected in a dry tube for blood chemistry and a urine sample (4 mL) was obtained from the first morning urination. Microalbuminuria was determined employing a sandwich immunoenzymatic quantitative assay, with a cutoff value of <20 mg/mL (UMELISA, Immunoassay Center, Havana, Cuba).

The variable “indicator of subclinical vascular damage” comprised the following variables: intima media complex (IMC) thickening and/or plaque, degree of retinopathy and microalbuminuria, and was dichotomized as follows: Slight damage (minor): patients without retinopathy or grade I retinopathy, and/or IMC thickening and/or plaques, microalbuminuria ≤ 20 mg/L. More severe damage (major): grades II or III retinopathy, IMC thickening and/or presence of plaques and microalbuminuria > 20 mg/L. Individuals with at least two altered parameters were included.

Data analyses:

χ^2^ test and t-student test were used to evaluate the associations between categorical variables, and to compare continuous variables respectively. A multiple regression analysis was performed to determine the association of MRI variables with indicators of vascular damage in HT. Statistical processing was carried out with the STATISTICA 10.0 program for Windows and the level of significance was estimated at *p* <0.05.

Ethical aspects:

The ethical principles for medical research in humans of the Declaration of Helsinki of the World Medical Association 2013 [39] were fulfilled. The individuality of the participants in the research was respected; safeguarding their privacy and the confidentiality of information, with the aim of minimizing the consequences on physical and mental integrity. All the subjects involved provided their informed consent in writing for their participation in the study, which was approved by the ethics committee of the Institute of Neurology and Neurosurgery. The investigation is part of the project 0404096 of the Cuban Minister of Health.

## 3. Results

We studied 157 patients between 28 and 74 years of age, 120 were HT and 37 were non-HT (Table 1). Patients older than 55 years comprised 57.5% of HT cases and 47.4% of non-HT cases. There was no significant difference between the groups studied respect to ages and sex of the patients, whereas the values of systolic and diastolic BP between HT and non-HT groups were significantly different. BP above 140/90 was present in 65% of HT patients. Taking into account the laboratory variables, only glycemia and cholesterol showed statistical differences between the groups. Intimal thickening was statistically higher in HT than in non-HT patients. The majority of HT patients (67.5%) presented no retinopathy or low-grade retinopathy (grades 0–I), and higher grades of retinopathy (grades II–III) were observed in 32.5%. Dyslipidemia was present in 60.8% of HT, which was significant in relation to non-HT patients (27%, *p* = 0.00). Meanwhile, the percentage of individuals with more than 3 risk factors was significantly higher in HT (54.2%) compared with non-HT subjects (10.8%).

In relation to the MRI variables, the majority of the findings related to CSVD were more frequent in HT patients (Table 2). In general, the group of HT patients presented a significantly greater number of white matter lesions (Fazekas modified grade 0–I), whereas only one non-HT subject showed grade II in this classification. In turn, Virchow–Robin perivascular spaces, lacunar infarcts, and microbleeds were more prevalent in HT than in the non-HT group. Although, the CSVD score between 0 and 1 was frequent in both study groups, 97.3% of the non HT subject had the lowest score and 45% of the HT patients had CSVD 2–4.

Years of evolution of HT and SBP were significantly associated with CSVD severity; the means were higher in patients with more severe CSVD (severity 2–4 group) (17.4 years and 150 mmHg, respectively). Carotid morphological variables, specifically IMT and/or the presence of plaques, were statistically associated with CSVD severity in HT patients. Most of the patients without intimal thickening had a lower CSVD severity, but even in these cases, more severe CSVD may be present in a small proportion of subjects. Also, the highest severity of retinopathy (grades II–III) and the presence of microalbuminuria were more frequent in patients with more severe CSVD (Table 3).

**Figure 1:**
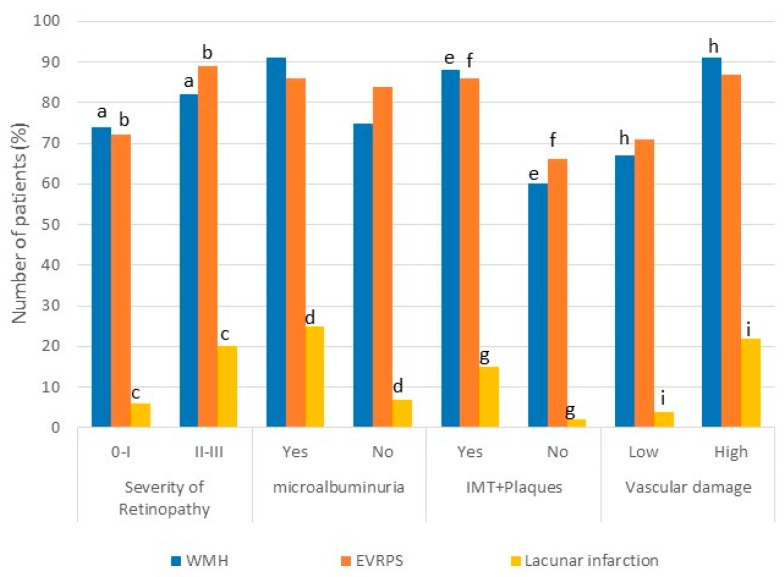
Association between imaging findings of CSVD and subclinical vascular damage indicators (carotid morphological variables, retinopathy, and microalbuminuria) in hypertensive subjects. WMH: White matter hyperintensities (Fazekas: Yes I–III/No: 0), Vascular damage (intimal thickening and/or plaques, retinopathy II–III, microalbuminuria > 20 mg/L). Same letters indicate significant difference (*p* < 0.05).

The presence of WMH (88%), EVRPS (86%), and lacunar infarcts (15%) were related statistically with greater thickness of the IMC and/or plaque (Figure 1) (*p* = 0.00, *p* = 0.01, *p*= 0.00, respectively). There was a significant increase of HT patients with more severe retinopathy (II–III) who had WMH (82%, *p* = 0.02), EVRPS (89%, *p* = 0.03), and lacunar infarct (20%, *p* = 0.01). Only HT patients with lacunar infarct were statistically associated with microalbuminuria (25% of patients, *p* = 0.03) (Figure 1).

Microbleeds were not significantly related with any of the indicators of vascular damage. This analysis was also performed with the non-HT group (excluding retinopathy severity), and the association of the indicators of vascular damage with the presence of hyperintensities and Virchow–Robin spaces was not significant (microalbuminuria: χ^2^ = 0.93 (*p* = 0.33); RF ≤ 3/>3: χ^2^ = 0.89 (*p* = 0.34); IMT and/or Plaque: χ^2^ = 0.03 (*p* = 0.85).

Linear regression multivariate analysis was performed considering CSVD as the dependent variable. Age groups ≤55 and >55 years, RF ≤ 3/>3, and the combination of vascular damage indicators (intimal thickening and/or plaques, retinopathy II–III, microalbuminuria > 20 mg/L) were considered as independent categorical variables, whereas SBP was a continuous independent variable. The model and intercept were significant. CSVD score was independently associated with the combination of vascular damage indicators, SBP and the interrelation between age groups ≤ 55/>55 years with vascular damage indicators (*p* = 0.02) (Table 4). The sensitivity of the combination of vascular damage indicators for detection of CSVD was 72.7% and the specificity was 58.2%. The efficiency of this marker was 66.1%.

**Figure 2:**
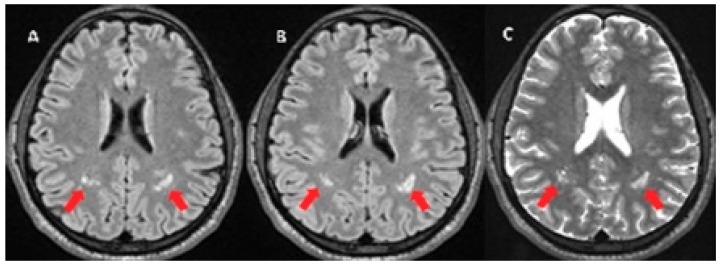
MRI with white matter hyperintensities (red arrow) in FLAIR (fluid attenuated inversion recovery) (**A**,**B**), and on T2 weighted images (**C**).

Figure 2 shows early damage in cerebral microcirculation with white matter hyperintensities in an asymptomatic male patient, 32 years old, with a history of 12 years with arterial hypertension. Fundoscopy revealed grade 2 hypertensive retinopathy. In addition, he presented subclinical renal vascular damage: microalbuminuria greater than >20 mg/L (29 mg/L) and despite pharmacological treatment, he maintained a high blood pressure (152/95).

**Figure 3:**
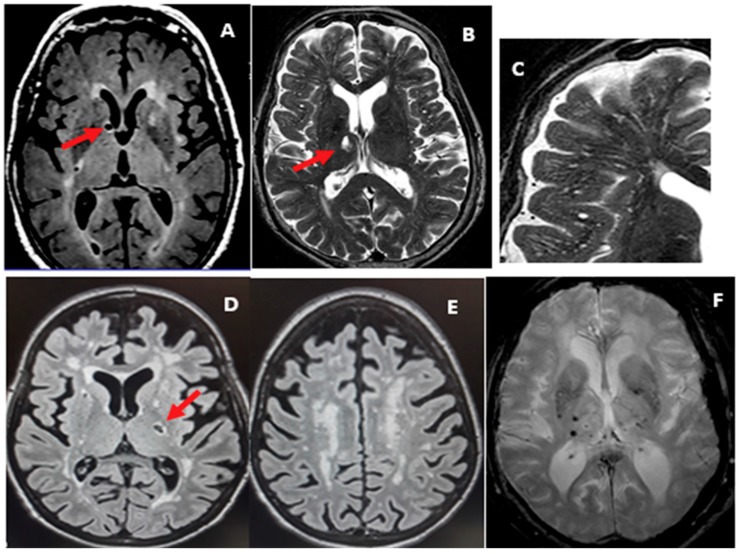
MRI, patients with cerebral small vessel disease and cognitive impairment/dementia (**A**–**F**). (**A**,**B**,**D**) lacunes (red arrow): are seen in FLAIR (Fluid Attenuated Inversion Recovery) as a cavity hypointense, with hyperintense halo, between 3 mm and 15 mm diameters (**A**,**D**) and on T2 as hyperintense signal similar to cerebrospinal fluid (**B**). Enlarged Virchow–Robin perivascular spaces (**C**) follow the course of a vessel through grey and white matter. White matter hyperintensities on T2-weighted images (**B**,**C**) and FLAIR (**D**,**E**), but without cavitation. The lesions are confluent around the ventricles and subcortical white matter (Fazekas III) and the signal differs from cerebrospinal fluid. (**E**) T2*-weighted MRI shows two microbleeds, areas of void signals in the right basal ganglia.

In contrast, Figure 3 shows several patients with cognitive impairment and disability who were excluded from the investigation. In these older patients, there is evident cerebral atrophy and multiple CSVD lesions.

## 4. Discussion

In this study, neuroimaging findings corresponding to CSVD were identified in asymptomatic hypertensive patients, which showed associations with the combination of subclinical vascular damage indicators (retinopathy grade, carotid Doppler variables, and microalbuminuria). Lacunar infarctions, microbleeds, and WMH (specifically modified Fazekas grade III) were only present in hypertensive patients. More severe CSVD score was observed in both groups (HT and non-HT), but in greater proportion in hypertensive patients. Associations between these cerebral parenchymal changes and intimal thickening and/or the presence of carotid plaques, retinopathy severity, microalbuminuria, SBP, years of evolution of HT, and the presence of cardiovascular risk factors were found. Additionally, CSVD score was associated with the combination of vascular damage indicators, which had a good sensitivity for the detection of CSVD in asymptomatic hypertensive patients.

The detection of white matter changes in neurologically asymptomatic hypertensive patients has distinctive characteristics: they appear earlier and more severely in these individuals compared with non HT subjects of similar age [30]. In this work we could verify this evidence, because WMH appeared in more than 50% of asymptomatic HT patients in the 5th decade of life, and with a high degree of severity (II–III) in almost half of these individuals. Similar findings have been described, in 44% of young hypertensive patients (mean age of 51.6 years) [22]. Some Cuban studies have found classic white matter lesions, which appear to be more frequent when the age of hypertensive patients increases [20,21,23]. Hernández-González et al. [23] described these alterations in 40% of individuals in a group of hypertensive patients with a mean age of 44.2 years; whereas in individuals with essential hypertension with a higher mean age (59 years) this alteration was observed in 73.9% [21] and then in a subsequent investigation in 70.6% [20]. It is evident that advanced age and HT are clinical risk factors associated with an increase in WMH in asymptomatic patients [24,40]. In addition, a relationship between high BP and lesions in cerebral white matter has also been demonstrated in several studies [30,41,42]. Park et al. [43] also studied these white matter changes by brain tomography in a group of neurologically asymptomatic subjects with no history of stroke, but with cardiovascular risk factors such as hypertension, DM, dyslipidemia, obesity, among others, and found a significant association between HT and the presence of white matter lesions [43]. WMH, of presumed vascular origin, have been ascertained as one of the most relevant markers of CSVD [32]. In fact, a study in Ecuador determined that silent lacunar and microbleed infarcts correlated with a worse cardiovascular health (determined by risk factors such as HT) [44]. In this sense, our findings concur with previous studies [43,44]. It is known that carotid vascular disease is related to the development of cerebrovascular events, as well as to CSVD [6,7,45]. Several studies demonstrated an association between IMT and WMH extension [45,46,47,48], as well as with the appearance of lacunar infarcts [35,49]. In contrast, Heliopoulos et al. evaluated hypertensive patients, and found no correlation between intimal thickening and the number of WMH [50]; however, this group demonstrated that a greater diameter of the common carotid artery (CCA) is associated with an increase in WMH after adjusting for clinical and demographic characteristics [50]. It is important to consider that a literature review in populations similar to ours, associating carotid doppler variables and the other CSVD imaging findings (lacunar infarcts and DVRS) were scarce and not very recent. In general, the association of intimal thickening and/or the plaque with the presence of lacunar infarctions, EVRPS, WMH, and their severity (Fazekas) is consistent with the possibility that the extracranial carotid damage is related to cerebral microcirculation. A set of cardiovascular risk factors lead to an increase in the presence of CSVD. In recent years, the prediction of cardiovascular morbidity risk has been an essential element in the guidelines for clinical practices for the prevention of these diseases, considering it a tool to establish priorities in primary care [51]. In this context, in the ADELAHYDE-2 study, this set of cardiovascular risk factors (advanced age, left ventricular hypertrophy by electrocardiogram, intimal thickening, and a high BMI) were positively related to the increase in WMH [47]. In our work, lacunar infarcts were related to the presence of three or more risk factors, in which advanced age, BMI, among other factors were included. Therefore, this could indicate that in hypertensive patients who have more associated risk factors there is a greater predisposition for white matter damage, reinforcing the concept that CSVD is a multifactorial process. Cerebral and renal blood vessels appear to exhibit a similar mechanism of adaptation in response to changes in BP [52]. Microalbuminuria has been found to be related with WMH in some diseases, including HT [53,54,55]. In our case the result was similar, since it was found to be associated with the severity of these lesions (Fazekas). In an investigation that included hypertensive and non-hypertensive patients without stroke, the presence of CSVD was associated with the increased risk of renal deterioration [OR 2.33 (95% CI 1.80–3.01)] compared to those subjects who did not present changes in brain parenchyma [52]. Once CVD has been established a positive association between renal dysfunction and early neurological deterioration is observed [56,57]. Although our patients did not have symptomatic CVD, some had lacunar infarcts, which were related to microalbuminuria. Therefore, exposure to high BP and other risk factors can originate small vessel disease [58], in which the entire vasculature can be affected, including the kidney, in a gradual and progressive manner.

High BP causes vasoconstriction and sclerotic changes in the retinal vessels, such as hyaline degeneration and intimal thickening, which causes arteriolar stiffness [59]. These changes can occur at the same time in the brain, affecting the function of cerebral arterioles in the control of focal blood flow [60]. In this study, hypertensive patients with greater retinopathy severity were associated with more EVRPS and lacunar infarctions, and greater severity of WMH. This result confirms previous reports, suggesting that hypertensive retinopathy is associated with an increased risk of coronary disease, CVD, and ventricular hypertrophy; hence, retinal changes should be taken into account as putative biomarkers for predicting cardiovascular disease [61,62].

The multivariate model allowed us to observe associations between indicators of vascular damage related to HT and CSVD. Other analyses, such as that of Yakushiji et al. [63], in which a population of neurologically asymptomatic individuals was evaluated, the maximum total score of the CSVD (including lacunar infarctions, microbleeds, WMH, EVRPS) was associated with the condition of hypertension [63]. In our study, we also found a significant independent relationship between SBP and this imaging marker, suggesting that SBP could have a predictive effect on cerebrovascular events. Similarly, another investigation described the relationship between the presence of WMH with higher values of SBP, DBP and average BP [64]. Meanwhile, in a population of asymptomatic hypertensive patients studied by Filomena et al. with characteristics similar to ours (where only lacunar infarcts and WMH were evaluated), they reported an association between white matter changes and SBP [65]. This could be explained, considering that high BP by itself facilitates changes in the microvasculature, by increased resistance, leading to white matter lesions [66]. In this regard, more research comparing the impact of HT on CSVD, taking into account other risk factors or not, is needed in order to define how much white matter is affected when adding cardiovascular risk factors.

The results suggest that indicators of subclinical vascular damage may be associated with CSVD, independently of other risk factors. Considering that microalbuminuria, intimal thickening, and retinopathy are markers of vascular damage as a common mechanism, changes in arterial resistance of hypertensive patients (vasoconstriction, intimal thickening remodeling, compliance changes, decrease in vasodilation, and rarefaction) have repercussions on global hemodynamics [66]. This study confirms the presence of CSVD in neurologically asymptomatic hypertensive patients. It might be affordable to perform early screening in these patients in order to avoid future CVD and cognitive impairment. However, non-neuroimaging markers would be more suitable, since MRI techniques are expensive and not readily accessible, even for high-income countries. In this sense, the combination of carotid morphological variables, microalbuminuria, and hypertensive retinopathy, as vascular damage indicators in asymptomatic hypertensive patients, seems to be sufficiently sensitive (72.7%) to detect CSVD in these populations. The specificity of this combination was lower (58.2%), but the efficiency was acceptable (66.1%). The main limitation of this study is the presence of concomitant comorbidities in the group of hypertensive patients which are also known to predispose to CSVD (DM, dyslipidemia, among others), and probably explains the low specificity encountered. This is practically impossible to evade, because purely hypertensive patients are very scarce, and the applicability of the results obtained with such a population would not be useful in medical practice. An adequate sensitivity of vascular damage indicator for the prediction of CSVD is useful for screening large populations. In this situation, hypertensive patients with positive vascular damage indicators would be flagged as subjects with a higher risk of asymptomatic CSVD. It should be borne in mind that amongst these there would be 41.2% of false positive individuals, according to the specificity obtained. This information could allow the physician to follow these patients more closely, or even have a brain MRI performed if it is deemed necessary.

## 5. Conclusions

The combination of carotid morphological variables, microalbuminuria, and hypertensive retinopathy seems to be an acceptable parameter to perform early screening and detect CSVD in asymptomatic hypertensive patients. It could be an inexpensive and available option to select those patients at higher risk, in order to treat them more intensely and prevent irreversible brain damage, which could lead to cognitive impairment, dementia, and stroke. This would be very important to preserve the quality of life and avoid the heavy financial burden on their families and society.

## Figures and Tables

**Table 1:** Characteristics of demographic, clinical, and laboratory variables in all patients.

Variables		HT *n* = 120	Non-HT *n* = 37	*t* (*p*)
Age, years, mean (SD)		55.7 (9.7)	57.7 (9.8)	−1.0 (0.27)
Patients > 55 years old, *n* (%)		69 (57.5)	23 (47.4)	χ^2^ = 0.32 (0.57)
Sex, female, *n* (%)		68 (56.7)	19 (51.4)	χ^2^ = 0.32 (0.57)
SBP, mmHg, mean (SD)		145.3 (20.9)	123 (11.6)	6.1 (0.00)
DBP, mmHg, mean (SD)		90.5 (12.7)	79 (6.9)	5.2 (0.00)
HT years, mean (SD)		14.8 (10.5)	-	-
HT with BP ≥ 140/90 mmHg, *n* (%)		78 (65)	-	-
BMI, kg/m^2^, mean (SD)		28.2 (4.8)	25.1 (2.1)	1.5 (0.14)
Creatinine, μmol/L, mean (SD)		83.5 (15.5)	83.3 (13.6)	0.1 (0.92)
Glycemia, mmol/L, mean (SD)		5.8 (1.5)	5.3 (1.2)	2.0 (0.04)
Cholesterol, mmol/L, mean (SD)		5.5 (1.1)	4.9 (1.1)	2.4 (0.02)
Triglycerides, mg/dL, mean (SD)		1.6 (0.8)	1.2 (0.7)	1.5 (0.14)
Microalbuminuria, mg/L, mean (SD)		13.8 (11.9)	8.4 (9.9)	1.6 (0.11)
IMT, mean (SD)		1.0 (0.19)	0.8 (0.2)	3.8 (0.00)
Retinopathy, *n* (%)	Grade 0–I	81 (67.5)	37 (100)	-
	Grade II–III	39 (32.5)	N/A	
Diabetes Mellitus, *n* (%)		30 (25)	7 (18.9)	χ^2^ = 0.58 (0.45)
Smoking, *n* (%)		45 (37.5)	9 (24.3)	χ^2^ = 2.17 0.14)
Obesity, *n* (%)		28 (23.3)	1 (2.7)	χ^2^ = 2.92 (0.08)
Dyslipidemia, *n* (%)		73 (60.8)	10 (27)	χ^2^ =12.6 (0.00)
FPB of CVD, *n* (%)		34 (28.3)	9 (24.3)	χ^2^ =0.22 (0.63)
>3 RF, *n* (%)		65 (54.2)	4 (10.8)	χ^2^ =21.6 (0.00)

HT: hypertensive patients; SD: standard deviation; SBP: systolic blood pressure, DBP: diastolic blood pressure, IMT: intima media thickness; FPB: Familiar pathological background; CVD: cerebrovascular disease; RF: risk factor.

**Table 2:** Neuroimaging findings of cerebral small vessel disease employing 3T brain MRI in HT and non-HT patients.

Variables, *n* (%)	HT *n*= 120	Non-HT *n*= 37	χ^2^ (*p*)
Lacunar infarcts	14 (11.8)	0	-
Microbleeds	5 (4.2)	0	-
EVRPS	93 (78.1)	17(56.7)	5.7 (0.02)
Modified Fazekas			
0–I	69 (57.5)	36 (97.3)	20.2 (0.00)
II–III	51(42.5)	1 (2.7)	
CSVD severity score			
0–1	66 (55)	36 (97.3)	22.6 (0.00)
2–4	54 (45)	1 (2.7)	

Modified Fazekas (hyperintense lesion of periventricular and subcortical white matter). HT: hypertensive patients. Non-HT: non-hypertensive patients. EVRPS: enlarged Virchow–Robin perivascular spaces. CSVD: cerebral small vessel disease.

**Table 3:** Relation between CSVD severity and carotid morphological changes, clinical, and laboratory variables in HT patients.

Variables	CSVD Low Severity (0–1) *n* = 65		CSVD High Severity (2–4) *n* = 55	t/χ^2^ (*p*)
Years of HT, mean (SD)	12.5 (8.3)		17.4 (12.1)	2.6 (0.01)
SBP, mmHg, mean (SD)	134.7 (19.4)		150 (19.9)	4.5 (0.00)
DBP, mmHg, mean (SD)	85.7 (11.6)		92.1 (13.5)	3.1 (0.00)
BP values, *n* (%)	<140/90 mmHg	28 (43.1)	14 (25.4)	4.1 (0.04)
	≥140/90 mmHg	37 (56.9)	41 (74.6)	
Risk Factors, *n* (%)	≤3	34 (52.3)	21 (38.2)	χ^2^ = 2.7 (0.10)
	>3	31 (47.7)	34 (61.8)	
Carotid morphological changes				
IMT, mean (SD)	0.87 (0.18)		1.04 (0.19)	5.3 (0.00)
IMC thickening and/or plaque, *n* (%)	Yes	27 (41.5)	45 (81.8)	χ^2^ = 20.8 (0.00)
	No	38 (58.5)	10 (18.2)	
Microalbuminuria > 20 mg/L, *n* (%)	9 (19.1)		15 (44.1)	χ^2^ = 5.9 (0.01)
Retinopathy, *n* (%)	Grade 0–I	50 (76.9)	31 (56.4)	χ^2^ = 6 (0.02)
	Grade II–III	15 (23.1)	24(43.6)	
Subclinical vascular damage indicators, *n* (%)	Minor	48 (73.9)	19 (34.5)	χ^2^ = 13.7 (0.00)
	Major	17 (26.1)	36 (65.5)	

SD: standard deviation; SBP: systolic blood pressure, DBP: diastolic blood pressure, IMT: intima media thickness; IMT: intimal media thickness; BP: blood pressure; Subclinical vascular damage indicators (intimal thickening and/or plaques, retinopathy II–III, microalbuminuria > 20 mg/L).

**Table 4:** Multiple regression analysis of CSVD variable in hypertensive patients.

Score of Cerebral Small Vessel Disease (Multiple r = 0.49; F = 3.57; *p* = 0.00)			
Variables	F	*p*	Beta (95% CI)
Intercept	5.44	0.02	-
Age groups ≤ 55/>55 years	1.79	0.18	−0.16(−0.39 0.08)
SBP	3.99	0.04	0.20 (0.01 0.40)
RF ≤ 3/>3	0.00	0.97	0.02 (−0.19 0.23)
Subclinical vascular damage indicators	6.3	0.01	−0.29 (−0.52 −0.06)
Age groups ≤ 55/>55 years–subclinical vascular damage indicators	5.66	0.02	−0.25 (−0.47 −0.04)

SBP: systolic blood pressure, HT: hypertension, RF: risk factors. Subclinical vascular damage indicators (intimal thickening and/or plaques, retinopathy II–III, microalbuminuria > 20 mg/L).
